# Electrical Stimulation of Regenerative Peripheral Nerve Interfaces (RPNIs) Induces Referred Sensations in People With Upper Limb Loss

**DOI:** 10.1109/TNSRE.2023.3345164

**Published:** 2024-01-16

**Authors:** Michael A. Gonzalez, Chinwendu Nwokeabia, Alex K. Vaskov, Philip P. Vu, Charles Lu, Parag G. Patil, Paul S. Cederna, Cynthia A. Chestek, Deanna H. Gates

**Affiliations:** Department of Robotics at the University of Michigan, Ann Arbor, MI, 48105 USA; Department of Robotics at the University of Michigan, Ann Arbor, MI, 48105 USA; Department of Plastic Surgery at the University of Michigan, Ann Arbor, MI, 48109 USA; Department of Plastic Surgery at the University of Michigan, Ann Arbor, MI, 48109 USA; Department of Neurosurgery at the University of Michigan, Ann Arbor, MI, 48109 USA; Department of Neurosurgery at the University of Michigan, Ann Arbor, MI, 48109 USA; Department of Plastic Surgery at the University of Michigan, Ann Arbor, MI, 48109 USA; Department of Biomedical Engineering at the University of Michigan, Ann Arbor, MI, 48105 USA; School of Kinesiology at the University of Michigan, Ann Arbor, MI, 48109 USA

**Keywords:** Sensory feedback, prosthesis, electrical stimulation, amputation

## Abstract

Individuals with upper limb loss lack sensation of the missing hand, which can negatively impact their daily function. Several groups have attempted to restore this sensation through electrical stimulation of residual nerves. The purpose of this study was to explore the utility of regenerative peripheral nerve interfaces (RPNIs) in eliciting referred sensation. In four participants with upper limb loss, we characterized the quality and location of sensation elicited through electrical stimulation of RPNIs over time. We also measured functional stimulation ranges (sensory perception and discomfort thresholds), sensitivity to changes in stimulation amplitude, and ability to differentiate objects of different stiffness and sizes. Over a period of up to 54 months, stimulation of RPNIs elicited sensations that were consistent in quality (e.g. tingling, kinesthesia) and were perceived in the missing hand and forearm. The location of elicited sensation was partially-stable to stable in 13 of 14 RPNIs. For 5 of 7 RPNIs tested, participants demonstrated a sensitivity to changes in stimulation amplitude, with an average just noticeable difference of 45 nC. In a case study, one participant was provided RPNI stimulation proportional to prosthetic grip force. She identified four objects of different sizes and stiffness with 56% accuracy with stimulation alone and 100% accuracy when stimulation was combined with visual feedback of hand position. Collectively, these experiments suggest that RPNIs have the potential to be used in future bi-directional prosthetic systems.

## I. Introduction

A GOAL of prosthetic research is to sufficiently replace the function of a hand through dexterous movement and naturalistic sensory feedback, both of which contribute to a sense of prosthetic embodiment [1], [2]. Current, commercially-available prostheses provide only incidental feedback, such as vibration of the motors [2], [3]. This feedback is insufficient to discriminate between different objects accurately [3] or to enable individuals to confidently lift objects without slips or drops [4].

The benefits of sensation have motivated the development of bi-directional prostheses that actively provide sensory feedback to the user. Several research groups have successfully delivered sensory feedback that is referred to the “phantom” hand of an individual with amputation by electrically stimulating the neural pathways that previously innervated the intact hand. Most commonly, researchers have stimulated peripheral nerves through extraneural cuff electrodes that wrap around residual nerves [5], or transneural electrodes that pierce a nerve [6] (see [7] review article for details on additional approaches). Using peripheral nerve interfaces, researchers have elicited sensations that are referred to the phantom hand and consistent in location and quality over several months [8], [9], [10]. Combined with sensorized prosthetic hands, stimulation of peripheral nerve interfaces enabled people with amputation to sense the stiffness and shape of virtual objects more accurately [11], improve their performance in tasks involving grasp control [9], and improve their ability to modulate grip force [8], compared to conditions where they did not have additional feedback. Home use of a sensorized prosthesis increased daily prosthetic wear time [12], quality of life [12], and embodiment [12], [13].

A frequent area of concern for peripheral nerve interfaces is the longevity of electrodes placed in or around the nerve. Indeed, implantation of cuff electrodes has caused abnormal morphology both at the nerve cuff level and in nerve branches distal to the cuff [14] and nerve inflammation [10]. Foreign body response to neural electrodes can cause nerve damage and mechanical trauma that can also affect the quality of signal recordings [15]. This can lead to increases in the charge required to elicit sensation [16], or result in the loss of functional recording electrodes [17]. However, several research groups have found that nerve cuffs [9] and transneural electrodes [16], [17] can elicit sensation when stimulated at similar stimulation parameters over several months [9], [16] to over a year [5], [17]. Importantly, within a single interface some active sites may be consistent while others are more unstable [16], possibly due to differences in surrounding tissue.

Here, we proposed to access sensory afferent pathways through electrical stimulation of regenerative peripheral nerve interfaces (RPNIs). RPNIs are biological constructs that are created through a surgical procedure in which a small piece of autologous muscle is wrapped around and sutured to an individually separated residual nerve fascicle. The muscle then revascularizes and the nerve reinnervates the muscle over a period of a few months [18]. Prior research has demonstrated that RPNI muscle tissue maintains a consistent size [19] and produces consistent motor signals when electrodes are surgically implanted into them after creation for periods of up to 3 years [20]. These same intramuscular electrodes can be used to electrically stimulate the construct. While not stimulating the nerve directly, as in prior approaches, RPNI stimulation likely results in direct afferent depolarization of free sensory nerve endings enclosed within the interface [21]. The approach of placing the electrode in muscle tissue rather than nerve also has the advantage of dampening electrode micromotions which contribute to mechanical trauma [15]. The muscle also acts to insulate the electrical signals, whereas in direct nerve approaches a polymer cuff is used to electrically insulate the nerve. We have previously demonstrated that stimulation of RPNIs can elicit sensations that are referred to an individual’s phantom limb as well as kinesthetic feedback [21]. While promising, sensory findings were limited to only two participants in only a few testing sessions.

The purpose of this work was to determine the potential for RPNIs to be used as a source of feedback for future bi-directional prosthetic systems. To be a functionally viable alternative to existing approaches, RPNI stimulation would need to produce sensations that are interpretable by the user, consistent over time, and graded such that the individual can feel multiple levels of feedback. Accordingly, we characterized the responses of four individuals with transradial amputation to electrical stimulation of their RPNIs over months to years. As our primary outcome, we quantified the amplitude of stimulation that each participant could detect (perception threshold) and the location and quality of the perceived sensation monthly. Secondarily, we quantified the amplitude at which sensation became uncomfortable (discomfort threshold), determined whether RPNI stimulation could provide participants with graded sensation, and whether graded sensation could improve a participant’s ability to identify object stiffness and size. Finally, we explored the effects of stimulating multiple RPNIs, simultaneously.

## II. Methods

### A. Participants

Four participants with transradial amputation participated in this study whose protocol was approved by the University of Michigan’s Medical School Institutional Review Board (IRBMED: HUM00124839; clinicaltrials.gov: NCT03260400). After providing their written and informed consent, participants had underwent surgery to have RPNIs created on their residual nerves and electrodes implanted into these RPNIs and residual muscles. Electrodes were custom bipolar percutaneous intramuscular electromyography electrodes (Synapse Biomedical, Oberlin, OH, USA). Each electrode had two contacts that were 10 mm and 5 mm in length, spaced 5 mm apart with a diameter of 0.75 mm. In some cases, multiple RPNIs were created on a nerve following intraneural dissection (denoted −1,−2, etc.). Details on how RPNIs are surgically constructed can be found in [18].

**Fig. 1.**
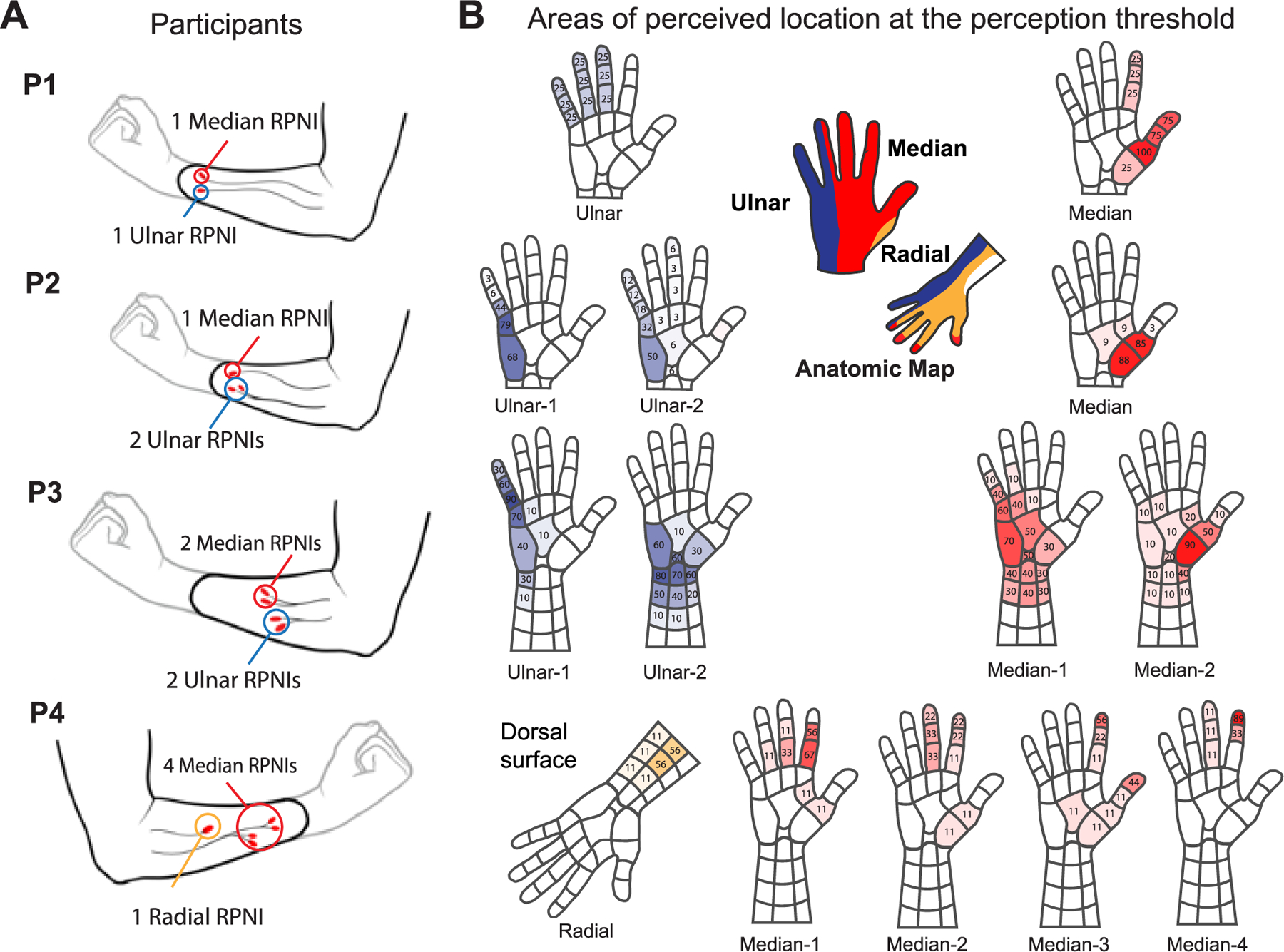
A) Surgical details for participants 1 (P1), 2, 3, and 4 regarding side of amputation, approximate length of amputation, and which RPNIs were implanted with electrodes for the purposes of stimulation and control signal acquisition. B) Areas and qualities of reported cutaneous sensation for the four participants at or just above their perception threshold. All cutaneous sensation was described as ‘tingling’ in nature.

P1 was a 33-year-old man who sustained a traumatic amputation of his right arm through the wrist. At 2 years post-amputation, P1 had RPNIs constructed out of his median, ulnar, and dorsal radial sensory nerves to treat refractory neuroma pain and phantom limb pain. In a surgery 3 years post-RPNI construction (5 years post-amputation), electrodes were implanted into one median and one ulnar nerve RPNI (Fig. 1A), in addition to six residual forearm muscles (8 total).

P2 was a 51-year-old woman who underwent a partial amputation of her right hand after an intravenous extravasation injury. Postoperatively, she had minimal residual hand function and chronic, unresolved neuroma pain and phantom limb pain. As a result, P2 underwent a distal transradial amputation and creation of one median nerve RPNI, two ulnar nerve RPNIs, and one dorsal radial sensory RPNI. One year post-amputation, P2 had electrodes implanted into her median and two ulnar RPNIs, in addition to five residual forearm muscles (8 total).

P3 was a 72-year-old man who underwent a transradial amputation of his right arm due to bone cancer and creation of two median nerve RPNIs, two ulnar nerve RPNIS, and one radial nerve RPNI. At 3 years post-amputation and RPNI construction, wires were implanted into his two median and two ulnar RPNIs and eight residual forearm muscles (12 total).

P4 was a 53-year-old man who underwent a transradial amputation of his left arm due to trauma. At the time of his amputation, he underwent targeted muscle reinnervation (TMR) prophylactically to prevent neuroma and phantom limb pain. During this procedure, the median nerve was split with one portion coapted to a motor branch of the flexor carpi ulnaris and one portion coapted to a motor branch of the flexor digitorum superficialis. Postoperatively, he developed severe neuroma pain which prevented him from wearing his prosthesis for more than 1–2 hours/day. About 2.5 years after his original amputation, he underwent revision amputation to treat his neuroma pain and to reduce the length of his residual limb. Intraoperatively, he was noted to have large neuromas-in-continuity of both fascicles of the median nerve at the TMR site consistent with the location of his severe neuroma pain. He underwent removal of the neuromas-in-continuity and creation of four median nerve RPNIs and one dorsal radial sensory nerve RPNI. In the same surgery, one bipolar electrode was placed into each of the five RPNIs in addition to seven residual forearm muscles (12 total).

Single day sensory thresholds and location of sensations for different stimulation parameters from P1 and P2 were reported on previously in [21].

### B. Stimulation Parameters

Stimulation was always delivered via symmetric, square, charge-balanced, biphasic waveforms with time-invariant stimulation parameters. Stimulation was performed in a monopolar configuration, with a grounding electrode placed on a bony landmark. Detection thresholds and sensitivity were determined by modulating stimulation amplitude.

### C. Functional Stimulation Range

A Digitimer-DS7a (Cephalon, Norresundby, Denmark) system stimulated RPNIs primarily using waveforms with a stimulation frequency of 20 Hz, pulse width of 100 *μ*s, and interphase interval of 10 *μ*s. Perception and discomfort thresholds were determined using a staircase method with adaptive step size, starting with a step size of 0.20 mA, followed by steps of 0.10 mA and 0.05 mA (Fig. 1B).

When determining the perception threshold, participants were asked “Do you perceive any sensation?” Stimulation amplitude steps were switched from incrementing to decrementing once the participant perceived sensation. These steps were switched back to incrementing and the step size was decreased when the participant no longer perceived a sensation. Once the smallest step size was reached, it was maintained until four reversals were recorded (switches from incrementing to decrementing, or vice versa). If more than eight presentations of the smallest step size did not result in a reversal, step sizes were increased back to 0.10 mA steps. The four final values of a successful thresholding were then averaged to yield the threshold value. If no sensation was perceived, pulse width was increased to 200 *μ*s, and the process was repeated. If no sensation was perceived at 200 *μ*s, or if sensation was not found after 10 steps following a reversal, stimulation was stopped and it was noted that no threshold could be determined for that session.

For each threshold, characteristics of elicited sensation were recorded, including quality and location of sensation. At the start of the study, participants reported this information verbally and by indicating the location using the experimenter’s hand. To improve the reliability of reporting, a piece of custom software was introduced to allow participants to draw the area of perceived location on a hand map using a touch screen computer. This was implemented after P1 had completed the study and P2 had completed 8 sessions. All perceived locations for P3 and P4 were recorded using the software. Perception thresholds were obtained approximately once a month.

Discomfort thresholds were characterized less frequently as they were, by design, uncomfortable for participants. Discomfort thresholding sessions were separated by approximately 1 month and used the same protocol as that used for perception thresholds, except that the participant was asked “Was the perceived sensation uncomfortable?” The participant was told that discomfort may be attributed to a variety of factors at their discretion, including sensations of pain or overwhelming intensity. We stopped stimulation if the participant experienced any in-loco sensation (within their residual limb rather than referred to the phantom limb) or involuntary contraction of the RPNIs. P1 and P4 had left the study prior to completing discomfort threshold testing. Finally, the functional stimulation range for each RPNI was calculated as the discomfort threshold minus the perception threshold.

We assessed the consistency of the perception thresholds over time using linear regression. We also assessed the stability of sensation location according to [16]. We first converted the hand maps to a grid where any sensation in that area was reported as ‘yes’ for sensation (Fig. 1B). For each RPNI, if the location reported was the same as the previous time (i.e. the same box in hand grid had a ‘yes’ on two consecutive visits), then the sensation location was considered as stable and given a score of 1, otherwise it was a 0. We then averaged the scores across all days tested. Sites with an average score greater than 0.6 were considered “stable”, those with scores between 0.3 and 0.6 were “partially stable”, and those with scores less than 0.3 were “unstable” [16].

### D. Sensitivity to Changes in Amplitude

The sensitivity of participants to changes in stimulation amplitude on a given RPNI was characterized using a two-alternative forced choice paradigm, similar to previous work [22], [23]. The reference amplitudes were chosen to be the midpoint of the functional stimulation range (i.e. 50%) and 25% between the perception and discomfort thresholds. Nine test amplitudes were selected, centered on the reference amplitude and separated by 5% of the functional stimulation range. This yielded experimental amplitudes of 5–45% and 30–70% along the functional range. Each set of nine test amplitudes was block randomized and presented either before or after the reference stimulus (determined randomly). The first amplitude was presented for 1.5 s of stimulation, followed by 1 s of rest, and finally by 1.5 s of stimulation with the second amplitude. The participant was instructed to report whether the first or second stimulus was more intense, ignoring any differences in area or sensation quality. This process was repeated until five blocks were completed, for a total of 45 stimulus pair presentations. After data collection, the data were plotted, with the test intensity along the X-axis, and the percentage of presentations in which the participant rated a test intensity as higher than the reference intensity on the Y-axis. A cumulative normal distribution was fitted to the data. The JND was then defined as the difference in stimulation amplitudes corresponding to the 50% and 75% probability intercepts on the fitted curve. JND measures were reported both in terms of nominal stimulation amplitudes and as percentages of the functional stimulation range. We then calculated Weber fractions from these JNDs, using

(1) K=ΔI/I,

where ΔI is the JND, I is the reference intensity used for the JND protocol, and K is the Weber fraction. A smaller K indicates greater sensitivity to a particular stimulus. Weber fractions were calculated as it is a common metric used in other papers that characterize sensitivity [22], [24]. As an alternative, a new metric called the percent of range was calculated, which normalizes the JND by the functional stimulation range rather than the reference intensity. Determining a JND required that a given RPNI have a defined functional stimulation range greater than 1 mA, and at least two observations of a discomfort threshold.

### E. Altering Stimulation Parameters

To characterize changes in sensation due to manipulation of stimulation amplitude and the number of stimulated RPNIs, participants were stimulated using a pulse width of 100 *μ*s and frequency of 20 Hz. Participants were asked to report their percept location and quality as stimulation amplitude increased in steps of 0.50 mA.

Simultaneous stimulation sessions were conducted using one of two stimulation systems: the NOMAD system (Ripple Neuro, Salt Lake City, UT, USA) or the Neuro Omega system (Alpha Omega, Alpharetta, GA, USA). The primary difference between devices was that the Neuro Omega system could stimulate at higher amplitudes (up to 7.5 mA) but could not guarantee phase-synchronization of the stimulation across multiple electrodes. The NOMAD system had a current limit of 2.54 mA, but could stimulate multiple electrodes in-phase. Here, a range of pulse widths (50 – 200 *μ*s), frequencies (10 – 50 Hz), interpulse intervals (25 – 100 *μ*s), and interphase (phase shift between the stimulation of each RPNI) delays (0 – 30 *μ*s) were explored. When stimulating, two RPNIs were selected at a time and amplitudes on both channels were incremented in steps of 0.20 mA until the participant reported sensation on one channel. That channel was then held at a constant amplitude while the other channel was incremented. If the percept on the first channel was no longer reported, it was subsequently incremented. This process was repeated until the participant could feel simultaneous sensation, or until the discomfort or safety thresholds were reached.

### F. Object Identification

We built and tested a closed-loop bi-directional feedback system using a prosthesis with built-in force sensors (LUKE arm, Mobius Bionics, Manchester, NH) and an electrical stimulation generator (Grapevine Nomad, Ripple Neuro, Salt Lake City, UT) to deliver biphasic square wave patterns to the median RPNI via a percutaneous bipolar electrode. The participant controlled hand open/close using a linear discriminant analysis (LDA) classifier built with EMG from all intramuscular electrodes (residual plus RPNI, except for the median RPNI). The EMG input signals were common-average referenced (the average of all signals was subtracted from each) and bandpass filtered between 100–500 Hz, and then the mean absolute value was extracted in 150 ms time bins with a 50 ms update rate.

The stimulator used for bidirectional control did not have sufficient range to vary amplitude alone (max amplitude = 2.54 mA). Instead, we chose to modulate both pulse width and amplitude to provide a wider range of charges. In the same experimental session as the object identification, we first found the perception threshold for pulse amplitude, holding pulse width at 400 *μ*s. We then fixed the amplitude at the perception threshold (0.5 mA) and varied the pulse width between 0 and 400 Hz, which was the maximal available pulse width. The perception threshold pulse width at this amplitude was 260 *μ*s. The participant did not express discomfort at any charge in the available range, so the discomfort threshold was the maxmial value (Amplitude = 2.54 mA and Pulse Width = 200 *μ*s.

Stimulation amplitude and pulse width ranges were mapped to force values using:

(2) $$PAstim=PA_{PT}+\frac{F}{F_{Max}}*\left(PA_{DT}-PA_{PT}\right),$$

(3) $$PW_{stim}=PW_{PT}+\frac{F}{F_{Max}}*\left(PW_{DT}-PW_{PT}\right),$$

where $PA_{stim}$, $PA_{DT}$, and $PA_{PT}$ refer to the pulse amplitude used for stimulation, the pulse amplitude at the discomfort threshold, and the pulse amplitude at the perception threshold, respectively. $PW_{stim}$, $PW_{DT}$, and $PW_{PT}$ refer to the pulse width used for stimulation, the pulse amplitude at the discomfort threshold, and the pulse amplitude at the perception threshold, respectively. Finally, F refers to the current force measured by the thumb of the LUKE arm, while $F_{Max}$ represents the maximum thumb force reading.

One participant (P2) completed an object identification task with this system, where she identified four objects of two different sizes and stiffnesses. The objects included a small soft foam sponge (2.5 × 2.3 × 2.4 cm, approx. stiffness: 2.80 N/m), a small wooden block (2.5 × 2.4 × 2.4 cm), a large foam block (7.5 × 4.4 × 4.4 cm, approx. stiffness: 2.25 N/m) and a 3D printed block made of polylactic acid (8.1 × 4.4 × 4.7 cm). Experimental values for the above force-parameter equations can be found in Table VI. We confirmed that the stimulation intensities associated with maximal compression of an object were distinguishable by the participant prior to experimentation.

P2 first probed each of the objects with her intact hand to internalize their different properties. She then completed four sets of trials where she was provided three attempts to probe the object before selecting which of the four objects it was. After each response, she was asked how confident she was on a scale from 0–100%. The first block was a training block of 16 trials, in which P2 was told whether she was correct or not after each trial. The following three blocks each provided different types of feedback to help identify objects: 1) visual feedback of prosthetic hand position on a computer screen, 2) visual feedback plus stimulation of the median RPNI where intensity was proportional to measured force, and 3) RPNI stimulation only. Each experimental condition set consisted of 32 trials. The last condition was completed over two days due to time constraints.

## III. Results

### A. Location and Quality of Sensation over Time

**Fig. 2.**
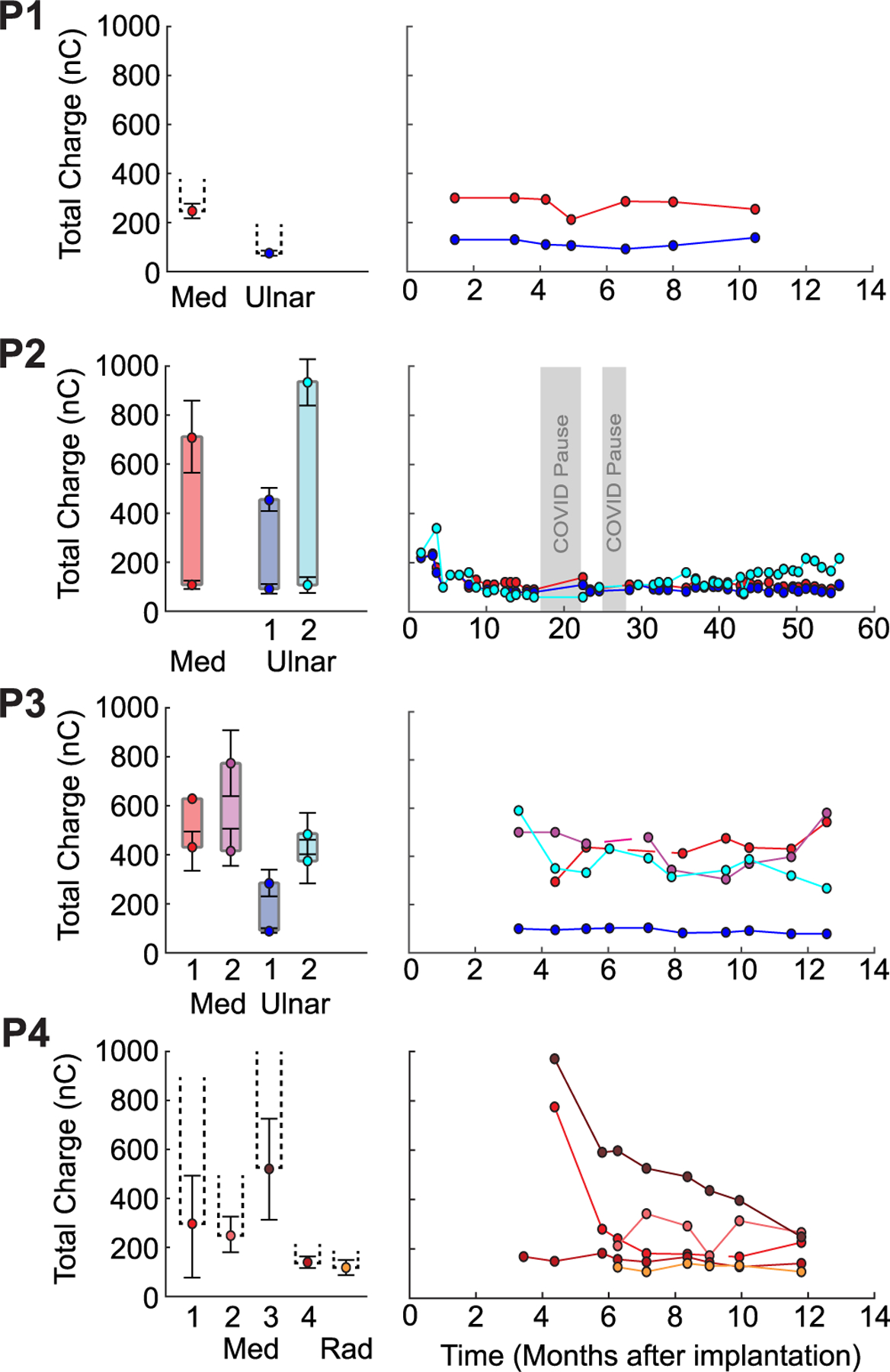
Left) Average and standard error of each participant’s perception and discomfort thresholds over time. Discomfort thresholds were only recorded for participants P2 and P3. For thresholds with less than three observations, only averages are presented. Right) Perception thresholds for each participant over time, measured in months after electrode implantation surgery. Dashed lines indicate gaps when a threshold could not be determined for a given RPNI.

P1 completed seven test sessions, starting 1 month after wire implantation (Fig. 2). Locations were recorded during four of these sessions. Stimulation of his Median RPNI resulted in cutaneous sensation in his thumb 75% of the time and index finger 25% of the time (Fig. 1B). The location of sensation in the thumb was stable (stability score > 0.66; Supplemental Materials S1). On two occasions, he also felt a sensation of thumb flexion. Stimulation of the Ulnar RPNI resulted in cutaneous sensation in the middle, ring, and small fingers each of which was only reported once throughout the four sessions. On one occasion, he also described “fluttering” or flexion of the little finger (Fig. 1B). Cutaneous sensations for both RPNIs were described as “tingling”.

P2 completed 43 sessions, beginning 1.5 months after wire implantation, with two pauses during COVID-19 shutdowns (Fig. 2). The areas and qualities of sensation were recorded during 34 sessions. Stimulation of her Median RPNI resulted in stable sensation at the base of the thumb 85–88% of the time (stability score: 0.71 – 0.84). Stimulation of the Ulnar-1 RPNI resulted in sensation along the ulnar border of the hand (68–79% of the time; stability score: 0.52 – 0.65 and in the small finger (3–44% of the time). Stimulating the Ulnar-2 RPNI resulted in “tingling” sensations in and around the small finger (12–18%), but also included “tugging” sensations in the middle (one occasion), ring (three occasions).

P3 completed 10 sessions, beginning 3 months after wire implantation. Stimulation of the Ulnar-1 RPNI resulted in stable sensation throughout the small finger (30–90%; stability score = 0.78) and ulnar side of the wrist (10–30%). Stimulation of the Ulnar-2 RPNI resulted in sensations across the base of the palm and stable sensations across the wrist/lower forearm (10–80%); stability score = 0.78. Stimulation of the Median-1 RPNI resulted in a wide area of sensations that were not expected anatomically. Sensation was frequently perceived as non-referred sensation within the residual limb (40% of the time). Stimulation of the Median-2 RPNI elicited sensation at the base of P3’s thumb 90% of the time (stability score = 0.89). In two instances, sensation was felt in his phantom wrist and once along the backs of all four of his fingers (not pictured). P3 reported all sensations as cutaneous and “tingling” in quality.

P4 completed 9 sessions, beginning 3 months after wire implantation. In the first session, only stimulation of the Median-4 RPNI consistently evoked any referred sensation. All RPNIs evoked sensation seven months after implantation. Stimulation of both the Median-1 and Median-2 RPNIs produced sensation in the index and ring fingers, typically along the adjacent edges of the two fingers. This sensation was only stable (stability score = 0.63) in the base of the index finger for Median-1. Stimulation of the Median-3 and Median-4 RPNIs produced partially-stable to stable sensation on the index finger (stability score = 0.50 and 0.75 for Median-3 and −4, respectively). On one occasion, stimulation of the Median-3 RPNI also evoked sensation on P4’s thenar eminence. Finally, stimulation of the Radial RPNI produced thin areas of sensation along the residual limb. P4 reported all sensations as cutaneous and “tingling” in quality. Of note, P4 experienced persistent ambient sensations in his phantom limb, including ‘burning’ sensations, intermittent ‘shocks’, and cramped fingers. Sensory reports took these into account before and after stimulation to ensure that recorded sensations only included novel sensations that resulted from stimulation.

### B. Functional Stimulation Range

The average perception threshold across all participants was 247 nC (range: 92 – 532 nC) (Fig. 2; Supplemental Material). Linear regressions of the perception threshold stimulation charge over time for most RPNIs were either decreasing (5/14 RPNIs, P2 Median-1, Ulnar-1, P3: Ulnar-1, Ulnar-2, and P4 median-3; *p* < 0.036), or were unchanging (8/14 RPNIs, *p* > 0.083). In one RPNI, the threshold increased over time (P3: Median-1, *p* = 0.045).

P1 and P4 did not complete discomfort threshold testing before exiting the study. P2’s discomfort thresholds were significantly higher than her respective perception thresholds, with a functional range of at least 3 mA across all three RPNIs (Fig. 2). In contrast, P3’s discomfort thresholds were only marginally higher than his perception thresholds. Additionally, P3 typically reported sensation at the electrode site prior to reaching a discomfort threshold. This, combined with difficulty finding consistent thresholds for P3, resulted in several sessions where no discomfort threshold could be determined for certain RPNIs.

### C. Sensitivity Testing

All three of P2’s RPNIs met the requirements for assessing just noticeable difference, as did the Median-2 and Ulnar-1 RPNIs of P3. Two of P3’s RPNIs were excluded, including his Median-1 RPNI, which had an inconsistent discomfort threshold, and his Ulnar-2 RPNI which had a functional stimulation range of 0.95 mA.

**Fig. 3.**
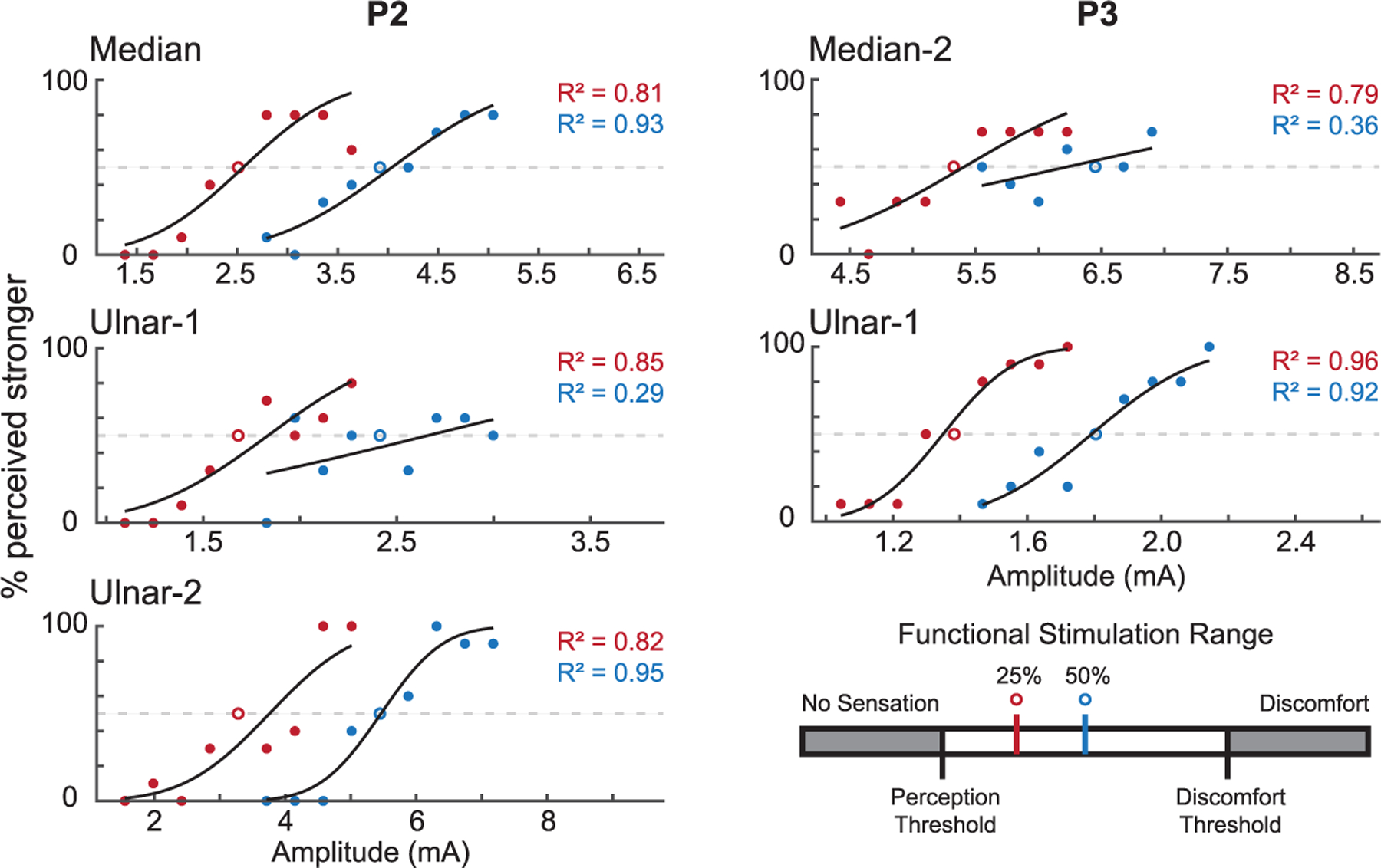
Results of two-alternative forced choice tasks for P2 and P3. Open circles indicate reference amplitudes, and are fixed at a value of 50% on the y-axis. Closed circles represent averages of ten trials for each test amplitude. Points were fit with cumulative normal distributions (CMDs). Data sets with a linear fit indicate that a CMD fit was not possible. (N: number of trials across all presented amplitudes, PW: pulse width, PF: pulse frequency).

Tests were centered around 25% and 50% of the functional range of each RPNI. For the 25% condition, both P2 and P3 were sensitive to changes in stimulation amplitude, as indicated by fits of cumulative normal distributions with inflection points between 0% and 100%) across each of their tested RPNIs (Fig. 3). Both participants were generally less sensitive to changes in stimulation amplitude (flatter cumulative normal distributions) during the 50% condition (Table II). For two RPNIs in the 50% condition (P2: Ulnar-1 RPNI, P3: Median-2 RPNI), the participants could not consistently differentiate between higher and lower stimulation amplitudes, resulting in poor fits (*R*^2^ < 0.4), with inflection points outside of 0% and 100%. In these cases, no JND could be determined (Fig. 3).

### D. Object Identification

**Fig. 4.**
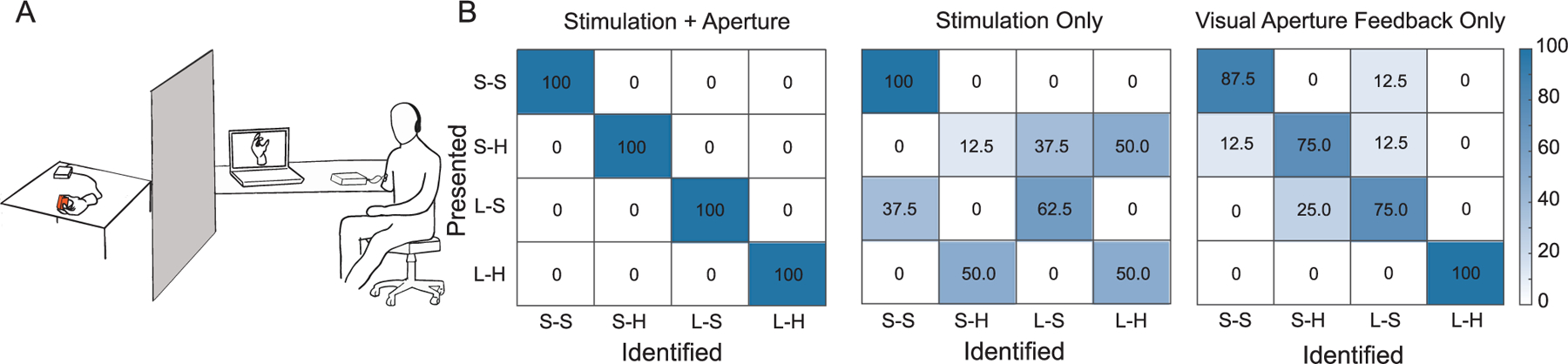
A) A depiction of the object identification task. B) Confusion matrices comparing the presented objects and the object the participant identified under three feedback conditions. S-S, S-H, L-S, and L-H represent the small soft, small hard, large soft, and large hard objects, respectively.

In the object identification task, P2 successfully identified all objects when presented with both visual feedback of a hand and stimulation of her median RPNI proportional to force (100%; (Fig. 4)). She also performed at higher than chance (25%) when provided with only stimulation (56%) or only visual feedback (84%). Confusion matrices indicate that she had more difficulty disambiguating object size compared to object stiffness with stimulation. The participant was similarly confident in identifying the objects between conditions (Stimulation only = 74%, visual only = 77.5%, visual+ stimulation = 83%).

### E. Influence of Stimulation Methods on Sensation

**Fig. 5.**
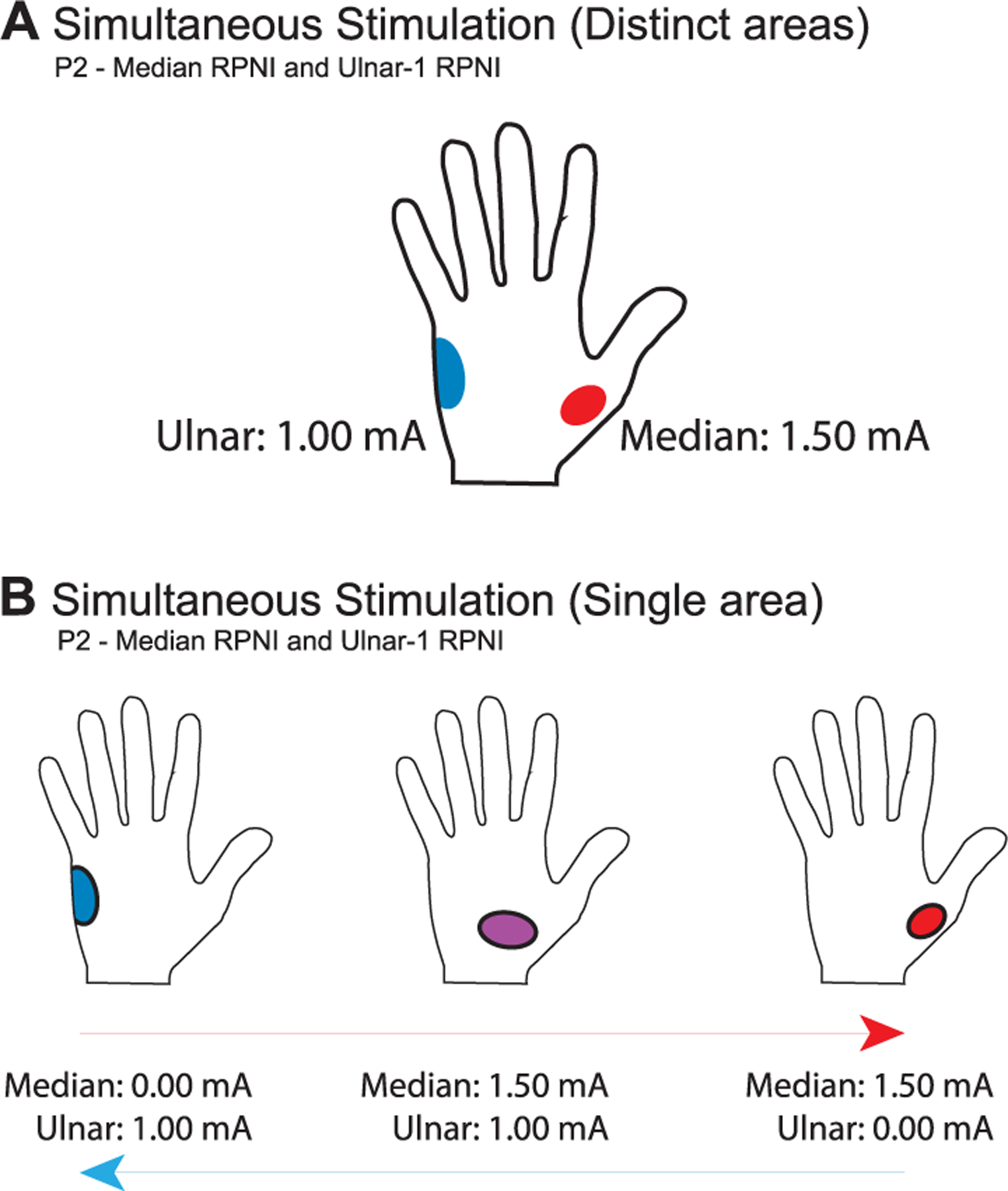
Experiments conducted with P2 to explore stimulation across multiple RPNIs. A) Simultaneous stimulation of P2’s Median and Ulnar-1 RPNIs typically produced two distinct areas of sensation. These two areas were approximately the same as the areas P2 reported when either RPNI was stimulated individually. B) Simultaneous stimulation of P2’s Median and Ulnar-1 RPNIs sometimes produced a single area of sensation that could shift position based on the relative stimulation amplitude of the two RPNIs.

In P2 and P3, we explored how sensory percepts changed in response to increasing sensation above the perception threshold. Generally, increasing the amplitude of the stimulation caused the sensation to become more intense, but did not change its location or quality. The only exception was P2’s Ulnar-2 RPNI, which changed in both area of sensation and quality of sensation as the stimulation amplitude increased (Fig. 5A). At 1.5 mA, just above the perception threshold, P2 reported a cutaneous tingling sensation along the ulnar side of her hand. The area of sensation increased with stimulation amplitude until 3.5 mA. At that amplitude, P2 additionally felt a “tugging” on her phantom ring finger. At 7.5 mA, P2 described a stronger tug on her ring finger and a lighter tug on her small finger, in addition to the previously reported cutaneous sensation. Further increases in amplitude only resulted in increases in intensity, but not any changes in the area or quality of sensation.

To determine how sensation changed during simultaneous stimulation of multiple RPNIs, an additional experiment was with P2 stimulating different pairs of RPNIs. Simultaneous stimulation of two RPNIs resulted in four different outcomes. For two RPNIs, A and B, which refer sensation to sites on the phantom hand *(a)* and *(b)*, either 1) the participant would feel sensation only at site *(a)*, 2) the participant would feel sensation only at site *(b)*, or 3) the participant would feel sensation at sites *(a)* and *(b)* (Fig. 5B). Which site the participant perceived seems to depend primarily on which RPNI was stimulated at a higher amplitude, or which generated the greatest intensity in sensation. Interestingly, if the participant experienced the two distinct areas of sensation and then the stimulation waveforms for each RPNI were shifted out-of-phase relative to each other, then the participant perceived only one of the two areas. This was repeatable for phase shifts of 3 *μ*s when stimulating the Median and Ulnar-1 RPNIs at their perception threshold.

Finally, in four of the seven sessions in which simultaneous stimulation was conducted, an additional behavior was observed: 4) the participant would feel a novel sensation *(c)* between sites *(a)* and *(b)*. In our study, this phenomenon was observed only when using one of our two electrical stimulation systems, and only for simultaneous stimulation of P2’s Median and Ulnar-1 RPNIs when both RPNIs were stimulated around their perception threshold (Fig. 5C). During sessions when this occurred, this novel point (c) was typically in the palm, close to the wrist and centered between the median and ulnar sides of the hand. Then, when the amplitude of stimulation to the Ulnar-1 RPNI was decreased, the perceived area shifted toward the median side. And when the amplitude of the stimulation the Median RPNI was decreased, the perceived area shifted toward the ulnar side. To test the consistency of this phenomenon, P2 was randomly stimulated either on the Ulnar-1 or the Median RPNI only, or simultaneously stimulated on both. In this experiment, P2 correctly identified that stimulation was directed at the left, right, or center of her hand in 27 of 30 trials (90% accuracy).

## IV. Discussion

An ideal bi-directional prosthesis requires a method of delivering somatotopically accurate sensory feedback that is consistent over time. Here, we demonstrated that electrical stimulation of RPNIs constructed on nerves in the residual arm could produce sensations that were referred to the phantom hand of participants with transradial amputation. The thresholds to elicit sensation were either consistent or decreased over 9, 54, 9, and 8 months for each participant for 13 of 14 RPNIs. The average perception thresholds for all RPNIs varied between 92 and 532 nC. In comparison, a recent review reported thresholds of < 50 nC for cuff electrodes, < 200 nC for FINE, < 100 nC (with most under 40 nC) for wire LIFE and tf-LIFE, < 40 nC for TIME, and < 20 nC for USEA [25]. The higher thresholds for RPNIs are likely due to the higher impedance of the muscle grafts used in the construction of RPNIs compared to the direct nerve stimulation of other techniques.

The high variability in sensory thresholds across RPNIs may result from a number of different factors. First, it is possible that the intrafascicular dissection of a peripheral nerve resulted in one fascicle that was predominantly made up of motor axons and another that was predominately made up of sensory axons. This becomes increasingly more likely as intrafascicular dissections of mixed motor-sensory peripheral nerves are performed. We expect that if an RPNI was predominantly composed of motor axons, then that RPNI would be less useful for evoking sensation, either requiring much higher currents, producing inconsistent sensation, or not producing any sensation at all. This may contribute to our findings for P3’s Median-1 RPNI, which consistently produced strong efferent motor action potentials for movements of the phantom thumb and index finger, but required high charge (436 nC) to evoke sensations at inconsistent locations. Additionally, this RPNI was the only one to have an increasing perception threshold over time. In addition, there was no perception threshold measured on three of 10 occasions. In P3, the RPNI’s were created at the distal transradial level where the median nerve may have already sorted into one predominantly motor fascicle and one predominant sensory fascicle. In contrast, splitting P2’s ulnar nerve and P4’s median nerve enabled sensation to be elicited in more specific areas (Fig. 1. Given the small number of participants with varied etiologies, it is difficult to determine an ideal number of fascicles for eliciting sensation in each unique nerve at each unique anatomic level.

It is also possible that differences in perception thresholds arose from the placement of the single bipolar electrode implanted into each RPNI. The density of sensory axons is not uniform throughout the RPNI and thus some electrodes may have been placed in regions of the RPNI that had more sensory afferents, thus requiring a lower threshold. To address this issue, future systems could consider use of a multi-sensor array. It is also possible that timing of electrode placement in the RPNI may have contributed to variability in the stimulation threshold. In three of four participants, electrodes were placed in pre-existing mature RPNIs, while in one participant (P4), the electrodes were placed at the same time as the RPNIs were created. The results measured from P4 suggest that it may take longer than three months for sensory axons to reinnervate the tissue and more than 6 months until perception thresholds stabilize (Fig. 2).

A majority of RPNIs elicited sensations in anatomically expected locations whose consistency varied from unstable to stable. Here, stability of a location was defined according to a stability score (adapted from [16]). While this measure does depend largely on how location was defined, it provides a way to quantify the consistency of sensation. More consistent, referred sensation may decrease the misalignment an individual with amputation perceives between their prosthesis and phantom limb, which can affect prosthetic ownership [1] and potentially improve prosthetic function. Importantly, in those cases where participants perceived sensation in locations that did not match anatomical expectations, sensations were partially-stable to unstable (e.g. P3’s Ulnar-2 and Median-1) and required greater amplitude to elicit. The high amplitude required in these cases could suggest that the perceived sensation was due to leakage into adjacent muscles or nerves, rather than from referred sensation via the targeted RPNI. Notably, other studies that used cuffs or intraneural electrodes for stimulation have also reported sensations in areas outside of the stimulated nerve’s receptive field [26], [27], [28], [29].

An additional goal of bi-directional prostheses is to provide graded sensory feedback on pressure or hand position. Our study found that participants were sensitive to changes in stimulation amplitude across multiple RPNIs. As done here, prior work has quantified the just noticeable difference (JND) in stimulation parameters using two-alternative forced choice tasks [22], [24], either reporting the JND alone or in addition to the Weber fractions (WFs). WFs roughly describe the fraction of a reference intensity that an individual can detect, with smaller WFs representing greater sensitivity. WF for RPNIs were between 0.08 and 0.21. In comparison, Gracyzk et al. [22] reported WF for median nerve cuff stimulation between 0.30 and 0.33 when modulating stimulation frequency and WF of 0.05 when modulating pulse width. Another study stimulating the tibial nerve intraneurally reported WFs between 0.038 and 0.057 when modulating stimulation amplitude [24]. Due to differences in participants, technologies, and methodologies it is difficult to directly compare between these findings.

In a case study, we also found that graded stimulation of RPNIs could be used to distinguish object properties. Using referred sensation proportional to force at the end of the prosthetic thumb when in contact with the object, P2 successfully identified a small, soft object 100% of the time and was able to identify all other objects at significantly above chance (25%) (Fig. 4). The small, hard item was most often misidentified as large and soft, possibly due to similar sizes and sensations when the large, soft item was compressed. The large, soft item was also frequently misidentified as small and soft. Object identification accuracy was 100% for each object when aperture was available in addition to force feedback. Similarly, others have shown that having both tactile and proprioceptive feedback is beneficial in identifying object properties [30], [31]. Future systems could also map aperture information to a different channel (i.e. stimulate a different RPNI) to improve performance if no RPNI provides appropriate kinesthetic sensation. This would be helpful in situations where the person does not have full view of the hand. In this application the RPNI we stimulated to induce referred sensation was excluded from the prosthetic control classifier. This was possible as the classifier was only actuating a single degree of freedom (i.e. opening and closing the hand), which could be predicted via multiple channels. For more complex control, such as individual finger and thumb motion, inclusion of the RPNI signals in the classifier substantially improves classification accuracy [32]. In this case, it may be preferable to use the same RPNI as both a control input and target for stimulation. At low enough stimulation frequencies, we expect that there would be sufficient time to deliver a stimulation pulse, let activity on a channel settle, and then record efferent motor signals from the same channel. This time frame is unknown, however, and is an important area for future research.

We also conducted a series of exploratory studies varying stimulation parameters and simultaneously stimulating multiple RPNIs. Our results demonstrate that increasing stimulation amplitude could expand the area of perceived sensation and/or change stimulation quality. As noted in a recent literature review, expansion of the area of sensation is common across stimulation approaches (e.g. nerve cuffs, intrafascicular electrodes) [7]. Changes in quality have also been previously reported, though not as frequently [33], [34]. These changes are likely due to the recruitment of additional nerve fibers. Also similar to prior work [26], [35], [36], we found that simultaneous stimulation of two electrode contacts resulted in two distinct areas of sensation (Fig. 5A). A few papers have reported novel sensation areas during simultaneous stimulation that were not reported during single-site stimulation [36], [37]. Scarpelli et al. [37] explained this new sensation location as resulting from a *tactile phi phenomenon*, which describes the perception of a phantom stimulus that lies between two adjacent, actively stimulated points. Shifting from primarily stimulating point A to primarily stimulating point B could then, in turn, be perceived as an *apparent movement sensation* from A to B. This apparent movement sensation was documented in participants stimulated using transcutaneous electrical nerve stimulation (TENS) [37], and our findings indicate that the same or similar mechanisms can be replicated via stimulation of RPNIs to produce apparent movement(Fig. 5B). This effect was only present in our participant when the waveforms sent to each RPNI were in-phase. Shifting the waveforms out of phase, increasing stimulation amplitude above the perception thresholds, increasing pulse width, and increasing frequency all resulted in evoking two distinct areas of sensations rather than one. While the ability to produce novel sensations without the need for additional electrodes would be beneficial for future systems, more work is needed to determine if this effect can be achieved in other individuals, and consistently over time.

This study was limited by the small and varied sample across experiments. Each participant was involved in motor experiments in addition to sensory experiments and some had limited availability each month. We prioritized perception threshold experiments and added other experiments as time permitted. Accordingly, some participants did not complete certain experiments prior to exiting the study. Future studies should assess sensitivity to stimulation parameters, multielectrode stimulation, and object identification to determine if these findings generalize to the broader population.

## V. Conclusion

This study demonstrated that electrical stimulation of RPNIs in individuals with upper limb amputation produced sensations that were referred to the phantom hand. For a majority of RPNIs, sensation was perceived at a consistent stimulation amplitude over months to years, and in consistent locations with consistent qualities. Participants were also sensitive to changes in stimulation amplitude, and could use this sensitivity to identify objects of different sizes and stiffnesses. Finally, we were able to simultaneously stimulate two RPNIs to generate either two distinct sensations, or one novel sensation that could be moved across the phantom hand. Future work will determine whether these findings apply to the broader population with limb loss and whether RPNIs can be used for simultaneous prosthetic control and feedback in functional tasks.

## Supplementary Material

Supplement 2

Supplement 1

## Figures and Tables

**TABLE I:** Participant Demographics

Participant ID	Age at implantation (yrs)	Sex	Etiology	Number of implanted RPNIs		
				Median	Ulnar	Radial
P1	33	M	Trauma	1	1	0
P2	51	F	Infection	1	2	0
P3	72	M	Cancer	2	2	0
P4	53	M	Trauma	4	0	1

**TABLE II:** Sensitivity to Changes in Amplitude

	P2						P3			
	Median		Ulnar-1		Ulnar-2		Median-2		Ulnar-1	
Range (mA)	1.10–6.74		0.95–3.88		1.12–9.77		4.30–7.75		0.96–2.86	
Reference	25%	50%	25%	50%	25%	50%	25%	50%	25%	50%
JND (mA)	0.52	0.64	0.34		0.70	0.49	0.64		0.11	0.16
JND (nC)	52.0	64.0	34.0		70.0	49.0	64.0		11.0	16.0
Weber Fraction	0.134	0.163	0.204		0.213	0.09	0.120		0.079	0.091
% Range	9.2	11.3	11.7		8.1	5.7	14.1		6.5	9.7
R^2^	0.81	0.93	0.85	0.29	0.82	0.95	0.79	0.36	0.96	0.92

**TABLE III:** Stimulation Parameters for the Two-Alternative Forced Choice Protocol

ID	PA (mA)	PW (*μ*s)	PF (Hz)	IPI (*μ*s)	Duration (s)
P2	1.2–7.2	100	20	50	1.5
P3	1.0–7.2	100	20	50	1.5

* Device experience refers specifically to the time an individual has been using their current type of device (e.g. A 1 DoF Hand versus a multi-DoF hand) rather than overall prosthetic experience.

**TABLE IV:** Stimulation Parameters for Perception and Discomfort Thresholding

ID	PA (mA)	PW (*μ*s)	PF (Hz)	IPI (*μ*s)	Duration (s)
P1	0–10	200	20	50	1.5
P2	0–10	100, 200	20, 100	50	1.5
P3	0–10	100	20	50	1.5
P4	0–10	100	20	50	1.5

**TABLE V:** Stimulation Parameters for Exploratory Stimulation Experiments

	PA (mA)	PW (*μ*s)	PF (Hz)	IPI (*μ*s)	Interface Delay (*μ*s)	Duration (s)
Manipulating area	0–10	100	20	50	0	1.5
Simultaneous stimulation	0–5	50–200	10–50	25–100	0–30	0.5–2.0

* Simultaneous stimulation parameters are given per contact.

**TABLE VI:** Mapping Between Force and Stimulation Parameters During Object Identification Task

Object	Avg. force reading (N)	Avg. peak PA (mA)	Avg. peak PW (*μ*s)	Avg. peak charge (nC)
Small soft	12.75	1.2	323.4	398.8
Small hard	21.37	2.2	379.6	851.9
Large soft	14.53	1.5	325.2	480.6
Large hard	25.50	2.5	411.8	1045

## Appendix

See Tables III–VI.
